# Preliminary evidence for a Theory of Mind impairment in patients with Anxiety Disorders

**DOI:** 10.1192/j.eurpsy.2022.489

**Published:** 2022-09-01

**Authors:** G. Santarelli, M. Innocenti, V. Faggi, V. Miglietta, I. Colpizzi, F. Galassi, G. Castellini, V. Ricca

**Affiliations:** 1 University of Florence, Human Health Sciences, Firenze, Italy; 2 University of Florence, Human Health Sciences, firenze, Italy

**Keywords:** Anxiety, Empathy, Theory of Mind, Anxiety disorders

## Abstract

**Introduction:**

Theory of Mind is defined as the ability to understand mental states of other people, and is notoriously impaired in patients with Autism Spectrum Disorder. A growing body of evidence suggests an impairment of Theory of Mind in several other psychopathological disorders. However, only few studies have assessed Theory of Mind in patients with Anxiety Disorders (AD), addressing only patients with Social Anxiety Disorder.

**Objectives:**

We aimed to investigate the differences in Theory of Mind between patients with AD and Healthy Controls (HC).

**Methods:**

We enrolled 35 patients admitted in the Psychiatric Unit of Careggi with diagnosis of AD and 31 HC. We administered them: Zung Anxiety Scale (ZSAS), Empathy Quotient (EQ), Reading the Mind in the Eyes (RMET), and Faux Pas test (FP). A t-test for independent samples was performed to assess between-group differences.

**Results:**

Zung total scores proved to be significantly higher in patients (t(60)=4.375, p<0.001), while Empathy Quotient total scores (t(61)=-3.325, p=0.002), detection of faux pas in Faux Pas test (t(61)=-4.957, p<0.001), RMET total scores (t(63)=-2.269, p=0.031) were significantly higher in healthy controls.

**Figure:**
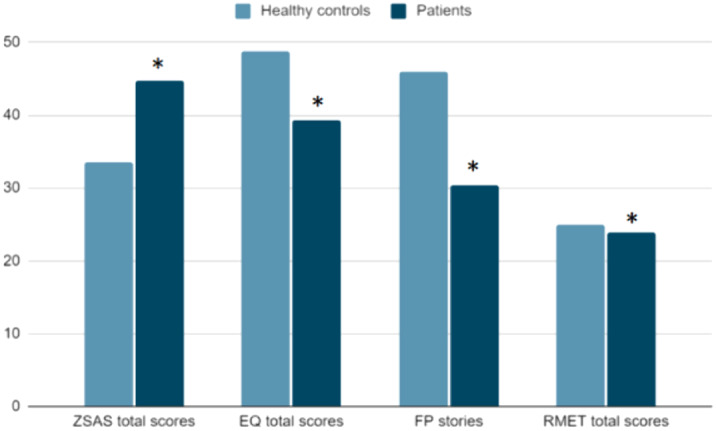

**Conclusions:**

Such preliminary data suggest impairment of Theory of Mind and Empathy in patients with AD as compared to HC. This could be linked to the development and maintenance of anxiety symptoms in patients with AD, making Theory of Mind a potential target in psychotherapy of AD.

**Disclosure:**

No significant relationships.

